# High genetic diversity and low differentiation retained in the European fragmented and declining Greater Spotted Eagle (*Clanga clanga*) population

**DOI:** 10.1038/s41598-019-39187-1

**Published:** 2019-02-28

**Authors:** Ülo Väli, Valery Dombrovski, Marina Dzmitranok, Grzegorz Maciorowski, Bernd-Ulrich Meyburg

**Affiliations:** 1 Estonian University of Life Sciences, Kreutzwaldi 5, 51006 Tartu, Estonia; 2 National Academy of Sciences, Academichnaia 27, 220072 Minsk, Belarus; 3 Institute of Zoology, Poznan University of Life Sciences, Wojska Polskiego 71C, 60–625 Poznań, Poland; 4 BirdLife Germany (NABU), POBox 33 04 51, 14199 Berlin, Germany

## Abstract

Characterising genetic diversity and structure of populations is essential for effective conservation of threatened species. The Greater Spotted Eagle (*Clanga clanga*), a large and globally vulnerable raptor, is extinct or in severe decline in most of its previous range in Europe. We assessed whether the remnants of European population are genetically impoverished, and isolated from each other. We evaluated levels of genetic diversity and population structuring by sequencing mitochondrial pseudo-control region and 10 introns from various nuclear genes, and estimated length diversity in 23 microsatellite markers. The European population has expanded since the late Pleistocene, and does not exhibit signs of a recent population bottleneck. The global genetic diversity in Europe was rather similar to that detected in other similar species. Microsatellites suggested shallow but significant differentiation between the four extant populations in Estonia, Poland, Belarus and Russia (Upper Volga region) populations, but introns and mtDNA showed that only the Estonian population differed from the others. Mitochondrial diversity was highest in the northernmost Estonian population, introns suggested lower diversity in Upper Volga, microsatellites indicated equal diversity among populations. A recent bottleneck was detected in Poland, which is consistent with the observed repopulation of the region. We conclude that significant gene flow and high genetic diversity are retained in the fragmented Greater Spotted Eagle populations; there is currently no need for genetic augmentation in Europe.

## Introduction

Human activities have caused the contraction and fragmentation of the distribution of many species. Small and geographically distinct remnant populations are often characterised by low genetic diversity, reduced growth, declines in numbers and increased extinction rates^1–5^. In the short term, inbreeding and genetic drift lower fitness of individuals and survival propbability of populations^2,6^, while in the long term populations that have lost their genetic variation have constrained evolutionary potential^7,8^. Hence, characterising genetic diversity and structure of populations is a research priority for threatened species because it can lead to more effective conservation and management strategies^8,9^.

Many populations of raptorial birds have declined due to habitat loss and fragmentation^10,11^. Moreover, persecution and environmental contamination have caused severe population constrictions^12,13^. These circumstances have led to genetic impoverishment of several species^14–20^, though there are also examples to the contrary: European White-tailed Eagles have retained genetic diversity despite a recent severe population bottleneck, which was probably short relative to that species’ long generational time^21^. Also, the effect of gene flow between populations of large, mobile animals is strong and can facilitate recovery of genetic diversity after a population constriction^22^. Indeed, although large raptors typically exhibit strong natal philopatry, which may favour genetic structuring, they also often range over large areas, and can overcome barriers like habitat fragmentation, which can promote increased genetic diversity. Moreover, individuals of migratory species may settle along migration routes^23^, which may further impede genetic structuring. To date, however, genetic studies have concentrated on non-migratory raptors, and the influence of migratory behaviour has remained poorly studied.

**Figure 1:**
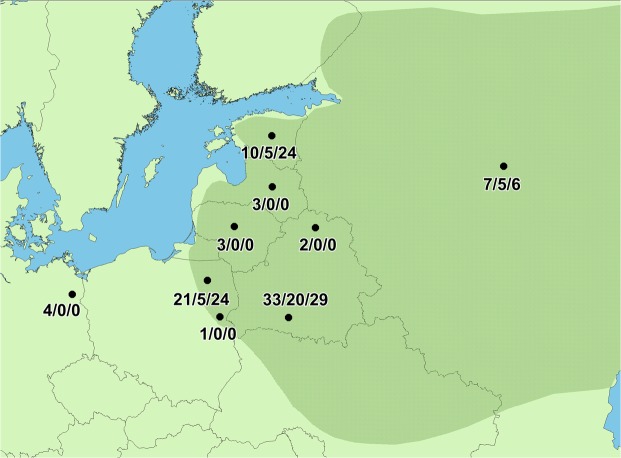
Sampling locations and numbers of samples analysed in this study (mtDNA/introns/microsatellites). Approximate European distribution range of the Greater Spotted Eagle is shaded.

The Greater Spotted Eagle *Clanga clanga* Pallas, 1811, is a large, long-lived, migratory raptor that breeds in temperate Eurasia, and winters in the southern parts of the continent and in Africa. Its population has been in continual decline for decades, and is considered globally Vulnerable and Endangered in Europe^24^. Although it is distributed from the Baltic Sea in the west to the Pacific Ocean in the east, this vast area is occupied by only a few thousand breeding pairs, with fewer than a thousand pairs breeding in Europe^24^. It has been extirpated throughout most of its historical European range, and the remaining populations are small and decreasing^10,24,25^ (Fig. 1).

At the end of the 20^th^ century the Greater Spotted Eagle was considered the least studied eagle in Europe^25^. Since then, however, several scientific studies have provided information that can aid its conservation. Although genetic methods have been used to detect its hybridisation with Lesser Spotted Eagle *Clanga pomarina*^26–28^, little attention has been given to better understanding the genetic architecture of its populations; genetic diversity has been explored within the Estonian population^29,30^, but large scale studies on population structure are lacking. Our aim is to fill this gap and explore the genetic structure and diversity of the Greater Spotted Eagle at the European level. We ask: (i) how diverse genetically are Greater Spotted Eagles across Europe, (ii) how much has fragmentation affected the genetic structure of the European population, (iii) how genetically isolated from one another are remaining populations, and (iv) have extant populations gone through a genetic bottleneck, which might have affected their evolutionary potential. The results will be examined within the context of Greater Spotted Eagle conservation, with an aim to provide advice on genetic management and look for potential opportunities for augmentation or translocation programmes.

## Results

### Genetic diversity

**Figure 2:**
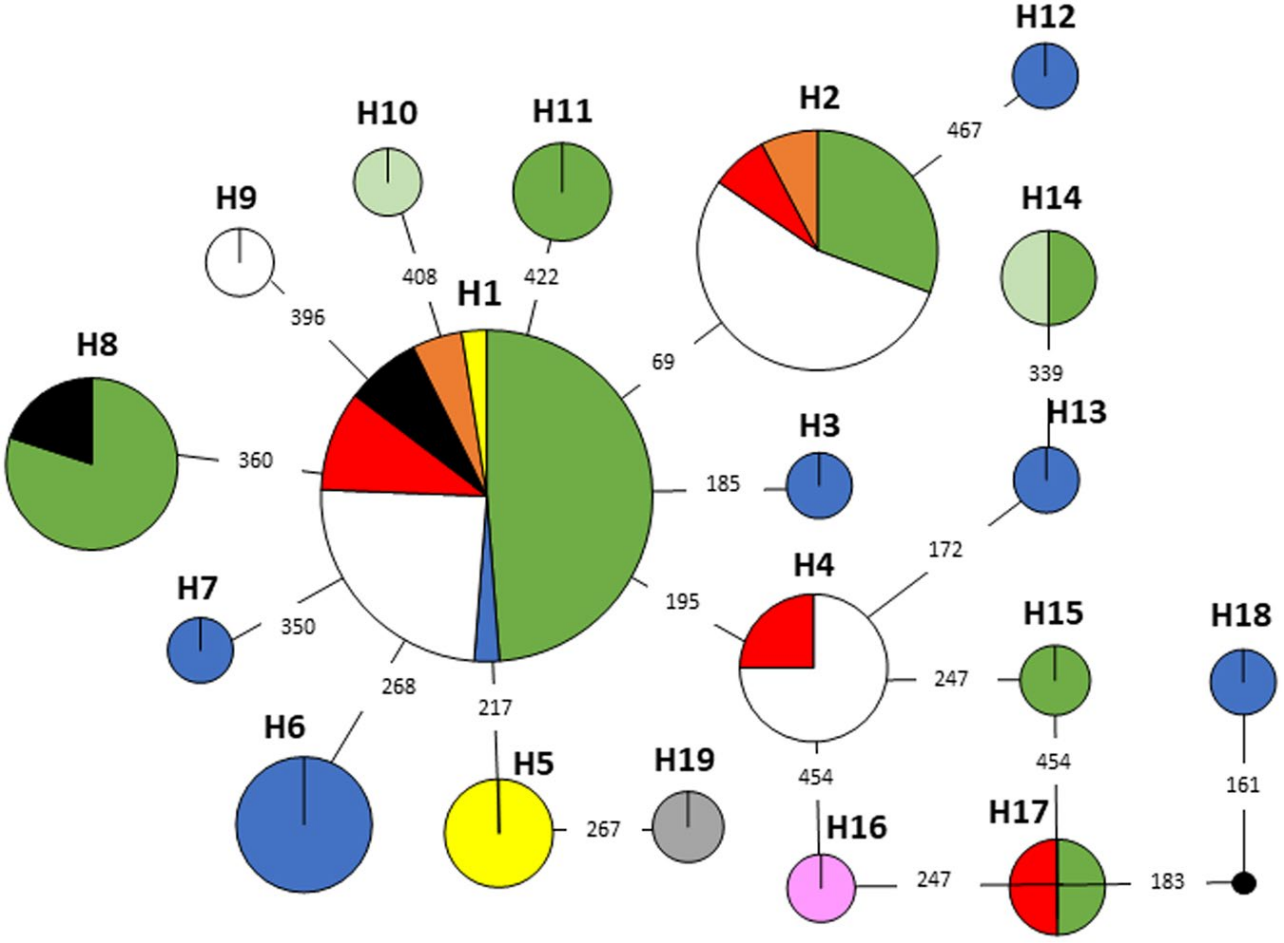
Median joining network of mtDNA haplotypes from Southern Belarus (green), north-eastern Belarus (yellow), Poland (white), Estonia (blue), Russia (red), Latvia (orange), Lithuania (dark green), Germany (black) and south-eastern Poland (pink). An Asian outgroup (Baikal region, Russia) is indicated as grey. Size of circles indicates the number of detected sequences in each haplotype. Indels were not included in the calculation of the network.

Among the 85 mtDNA sequences of 470 nucleotides studied, 19 mtDNA haplotypes were defined by 18 polymorphic sites (14 transitions and 4 transversions). Additionally, two single-nucleotide indels and a 26-nucleotide indel was found. There was one dominant haplotype containing 42 individuals, but it differed by only a single mutation from another haplotype with 13 individuals; other haplotypes contained five or less individuals (Fig. 2). The longest European branches were as much as five mutations from the main haplotype and up to seven mutations from the reference sample from the Baikal region (i.e. an Asian sample), which differed from the main European haplotype by only two mutations. The highest mtDNA haplotype and nucleotide diversities were found in Estonia, while in other populations the values were close to or below global values (Table 1).

**Table 1:** Mean values for measures of genetic diversity in four Greater Spotted Eagle populations: number of studied samples (*N*), haplotypes (*Nh*) and alleles (*Na*), haplotype diversity (*Hd*), nucleotide diversity (*π*), allelic richness (*Ar*) expected heterozygosity (*He*) and observed heterozygosity (*Ho*)., Garza-Williamson index (*M*).

	MtDNA				Introns							Microsatellites					
	*N*	*Nh*	*Hd*	*π*	N	*Nh*	*Hd*	*π*	*Ar*	*Ho*	*He*	*N*	*Na*	*Ar*	*He*	*Ho*	*M*
Belarus	33	7	0.62	0.0028	20	4.3	0.39	0.0008	2.71	0.32	0.40	29	4.83	2.21	0.55	0.47	0.88
Estonia	10	7	0.91	0.0056	5	2.5	0.38	0.0007	2.50	0.30	0.34	24	4.65	2.24	0.56	0.56	0.87
Poland	21	4	0.67	0.0017	5	2.7	0.42	0.0008	2.70	0.40	0.43	24	4.47	2.29	0.58	0.54	0.83
Russia	7	4	0.71	0.0028	5	1.5	0.20	0.0003	1.50	0.20	0.19	6	3.18	2.09	0.51	0.54	0.81
Global	85	19	0.74	0.0028	37	5.3	0.35	0.0006	2.61	0.31	0.38	83	5.82	2.27	0.53	0.47	0.85

In total, 134 alleles were detected in microsatellites, ranging between 2 and 13 per locus (mean 5.8). Mean values of allelic richness (*F*_*3,88*_ = 0.64; P = 0.58), expected heterozygosity (*F*_*3,88*_ = 0.50; P = 0.68) and observed heterozygosity (*F*_*3,87*_ = 0.75; P = 0.52) were very similar among the four populations (Table 1).

Intron sequences resulted in 54 alleles (haplotypes), but the mean number of alleles per locus across populations (2.7) was lower than in microsatellites. Populations in Estonia, Belarus and Poland exhibited rather similar values of diversity, and although diversity values for Russia were about two times lower, the difference was not significant (ANOVAs for haplotype diversity: *F*_*3,36*_ = 1.37; *P* = 0.27, allelic richness: *F*_*3,36*_ = 2.35; *P* = 0.08, expected heterozygosity *F*_*3,36*_ = 1.46; *P* = 0.24).

### Population differentiation

**Figure 3:**
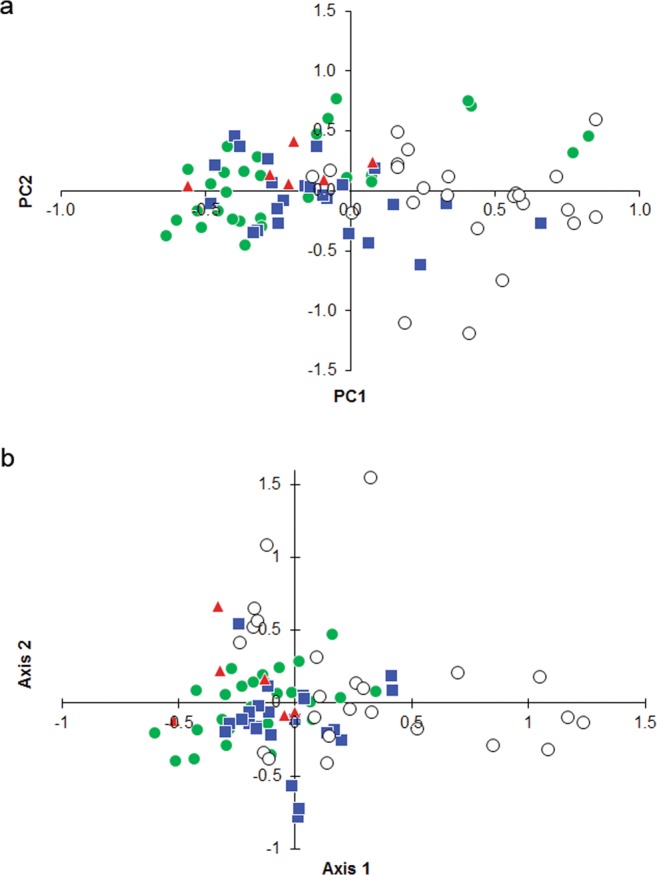
Spatial distribution of Greater Spotted Eagle microsatellite genotypes according to the (**a**) Principal Coordinate Analysis and (**b**) Factorial Correspondence Analysis. Genotypes from Belarus are presented as green circles, those from Estonia as blue squares, Poland as white circles and Russia as red triangles.

AMOVA suggested that most of the genotypic variation was found within individuals (80% by introns and 79% by microsatellites), less within populations (13% and 17%), and only a small fraction of variation was found among populations (7% and 4%). According to the mtDNA analysis above the individual level, 95% of variation was detected within, and only 5% between populations. The plots of Factorial Correspondence Analysis and Principal Coordinates Analysis of microsatellites suggested close relatedness between Belarussian, Estonian and Russian populations and distinctiveness of Polish population, but the distribution of plotted populations overlapped to a great extent (Fig. 3). Also, pairwise *F*_*ST*_ values indicated low differentiation between the studied populations (Table 2). However, results were inconsistent, with a few significantly high *F*_*ST*_ values detected with mtDNA and intron sequences, and many significantly low *F*_*ST*_ values with microsatellites (Table 2). Isolation by distance did not explain differentiation of populations (Mantel test for microsatellites: *R*^2^ = 0.17, *P* = 0.36, introns: *R*^2^ = 0.07, *P* = 0.40; Supplementary Fig. S1).

**Table 2:** Pairwise population differentiations (*F*_*ST*_) between Greater Spotted Eagle populations.

	MtDNA	Introns	Microsatellites
Belarus – Estonia	0.100**	0.043	0.012
Belarus – Poland	0.036	0.077	0.044***
Belarus – Russia	0.000	0.060	0.042*
Estonia – Poland	0.103**	0.144*	0.031***
Estonia – Russia	0.000	0.223*	0.036*
Poland – Russia	0.011	0.140	0.055*

Bonferroni-corrected significant differences are indicated by asterisks (*P < 0.05, **P < 0.01, ***P < 0.001).

**Figure 4:**
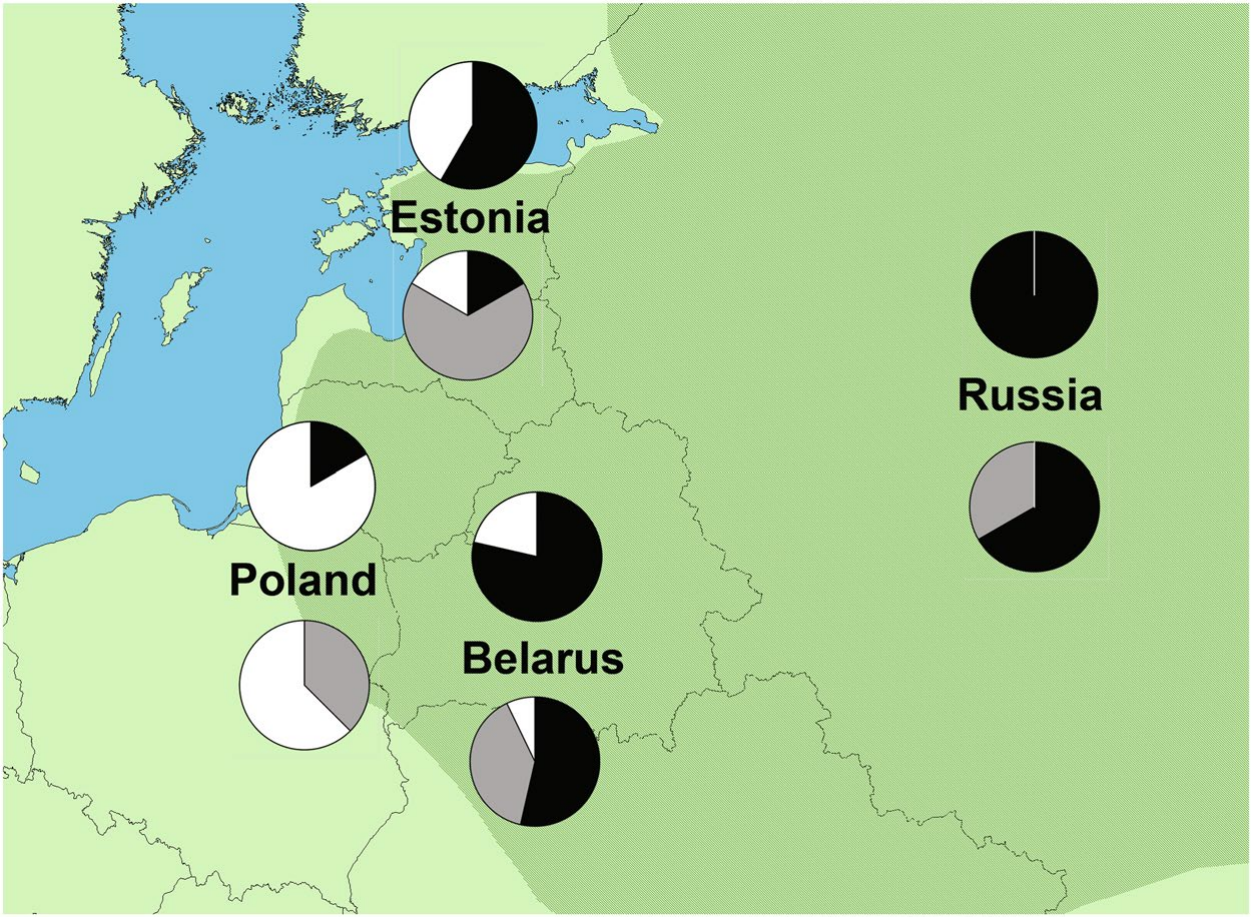
Proportions of Greater Spotted Eagle samples assigned in two clusters (upper pie-charts) or three clusters (lower pie-charts) in the four studied populations according to STRUCTURE analysis of microsatellites. Approximate European distribution range of the Greater Spotted Eagle is shaded.

In the STRUCTURE analysis of microsatellites, three groups (K = 3) were suggested by *LnP(D)* as at larger *K* values this parameter increased only slightly (Supplementary Fig. S2). *ΔK* suggested that individuals are most likely clustered into two or three groups (Supplementary Fig. S2). Both groupings (two or three clusters) revealed the east-west gradient in clustering of birds (Fig. 4). At *K* = 3, most Polish birds belonged to the first cluster, most Estonian birds to the second and most Russian birds to the third one, while the proportions of the second and third groups were nearly equal in Belarus (Fig. 4). The Polish population lacked the birds of the third cluster, and the Russian population the birds of the first cluster. Nearly equal values were obtained for *K* = 2, with a 70% cut-off criterion. In that case, the second group was replaced by birds having assignment probabilities intermediate between the two clusters, the only difference being that the proportions of the two groups was interchanged. Using *K* = 2 with the 50% cut-off value, all Russian and most Belorussian and Estonian birds belonged to one cluster, whereas most Polish eagles to the other. STRUCTURE analysis of microsatellites with prior information on source population (*K* = 4) detected no recent migrants in any of the populations (Supplementary Fig. S3). All individuals had high assignment values to their source population (q > 0.85), except two individuals in Belarus (q = 0.60 and 0.79) and one in Volga (q = 0.73). STRUCTURE analysis of introns was impeded by incomplete convergation and did not yield any detectable population structure. With *K* = 2, *q*_*max*_-values (highest assignment to a group) ranged between 0.50 and 0.71 (mean = 0.59) and with *K* = 3 between 0.34 and 0.61 (mean = 0.42). Similar results were obtained when introns and microsatellites were analysed in combination (*K* = 2: *q*_*max*_ = 0.58 (0.50–0.70); *K* = 3: *q*_*max*_ = 0.38 (0.43–0.50).

Median joining network did not reveal any clear structure among European mtDNA samples (Fig. 2). There was one main haplotype, incorporating most samples from nearly all sampled countries, surrounded by several other, large and small, haplotypes. The Asian reference sample formed a separate haplotype, but was only two mutations away from the main European haplotype. However, a star-like pattern was distorted by a long branch of seven haplotypes, up to five mutations distant from the main haplotype. Median-joining networks of mtDNA haplotypes in four larger populations were strikingly different. Star-like patterns characterised Belorussian, Polish and Russian populations, although not as clearly as at the European level; there were three distant haplotypes in Belarus and one in Russia, while samples were distributed rather equally among haplotypes in Poland (Supplementary Fig. S4). All samples from Estonia were distributed among distant haplotypes of low frequency (Supplementary Fig. S4). Mismatch distribution was bimodally distributed in the Estonian population, and unimodally in the other three populations.

### Population history

Mismatch distribution of mtDNA sequences was unimodal, but most haplotypes differed by one mutation from the main haplotype. For the global mtDNA sample, both Tajima’s D (−1.8, *P* = 0.008) and Fu’s Fs (−1.8, *P* < 0.001) were significantly negative, indicating population expansion. We obtained various estimates on the time of expansion, depending on the method and mutation rate used (Table 3). As the least-square procedure to fit expected and observed mismatch distribution did not converge after 2000 steps, these results should be taken with caution.

**Table 3:** Time of population expansion estimated using various models and mutation rates. 95% confidence intervals are given in brackets.

	Tau	Time (years, 6% mutation rate)	Time (years, 14.8% mutation rate)
Demographic expansion (ARLEQUIN)	1.25 (0.99–1.63)	44,326 (35,177–57,801)	17,970 (14,261–23,433)
Spatial expansion (ARLEQUIN)	0.73 (0.46–3.84)	26,064 (16,348–136,170)	10,566 (6,627–55,204)
Population expansion (DNASP)	0.88	31,277	12,680

No recent bottleneck was detected in European population, as the one-tailed probability for heterozygote excess among microsatellites was non-significant (*P* = 0.26), and the proportion of different alleles in frequency classes followed a typical L-shaped distribution (Supplementary Fig. S5). However, there was significant microsatellite heterozygote excess in Poland (one-tailed *P* = 0.005), suggesting a recent population bottleneck, although the proportion of different alleles in frequency classes was only slightly shifted (proportions in two lowest frequency classes were equal) from the L-shaped distribution (Supplementary Fig. S5). In other populations, probabilities for heterozygote excess were non-significant (*P* > 0.36), and the proportion of alleles in frequency classes followed the L-shaped distribution (Supplementary Fig. S4). Garza-Williamson index exceeded 0.81 in all populations, indicating no recent bottleneck.

## Discussion

The maintenance of genetic diversity is essential for supporting the survival probability of individuals and the evolutionary potential of populations^2,7,8^. Although there are examples of long-term survival of populations despite low genetic diversity^31,32^, correlation between genetic diversity and population ‘health’ is near-ubiquous^3,8^. This study indicates that genetic diversity has been retained in the Greater Spotted Eagle at the European level, and in the four studied populations, despite declines in numbers and extent during the 20^th^ Century^10^. Despite our use of the mitochondrial pseudo-control region, and not (as in most earlier studies) the control region, we obtained similar values for mitochondrial haplotype and nucleotide diversity to those recorded for several other species of bird of prey^18,19,21,33–36^. Results of microsatellite studies are often not easily compared because diversity values depend strongly on marker set selection. This is not the case in this study. Cross-species amplification has been rather successful in eagles, and several earlier studies have used the same (although variable set of) microsatellites used by us and obtained from White-tailed Eagle and two imperial eagle species (*Aquila adalberti*, *A*. *heliaca*). Because we did not analyse only the most polymorphic markers, but all available markers, our estimates could be somewhat lower than those from other studies. However, heterozygosities detected were not very different from imperial eagles^18^ or the White-tailed Eagles^21,37^. Still, interspecific comparisons should be taken with caution, because even in a population of the White-tailed Eagle population, various studies using different sets of microsatellites have resulted in different heterozygosity estimates^21,32,37^.

Typically, rare species are genetically less diverse than abundant species. This has been demonstrated in various organisms by comparing pairs of threatened and non-threatened sister species^3^. For example, Spanish Imperial Eagle *Aquila adalberti*, an endangered raptor with a confined and fragmented distribution, has significantly lower genetic diversity than its sister-species, the more abundant and widespread Eastern Imperial Eagle *A*. *heliaca*^18^. A similar difference has been noted between the endangered Madagascar Fish-eagle *Haliaeetus vociferoides* and African Fish-eagle *H*. *vocifer*^32^. Surprisingly, the rare Greater Spotted Eagle has higher mitochondrial diversity than the Lesser Spotted Eagle^29,30^, a closely related species with rather numerous breeding populations across most of its distribution range. However, the range of the Lesser Spotted Eagle, which breeds almost entirely in Central and Eastern Europe and to some extent the Middle East, is rather restricted when compared to that of the Greater Spotted Eagle, which is distributed across a vast area of Eurasia. This suggests that range size, and probably historical population size, plays a larger role in determining current genetic diversity than the current population size. Indeed, both Spanish Imperial Eagle and Madagascar Fish-eagle have very restricted ranges compared to their widespread sister species. Moreover, Madagascar Fish-eagle has persisted for a long time despite low diversity^32^. Also Red Kite *Milvus milvus*, a species with a relatively small breeding range, exhibits significantly lower genetic diversity than the widespread Black Kite *M*. *migrans*^19^.

Different diversities among sister-species may also arise from different patterns of colonisation, a feature that is more common in northern latitudes. During glacial times, the Greater Spotted Eagle population may have fragmented in multiple refugia, while the Lesser Spotted Eagle seems to be survived in only a single refugium^30^. One may suspect that the marginal populations studied by us would be atypically diverse, but this is probably not true; in an analogous study of the White-tailed Eagle, diversity values of peripheral populations were nearly a magnitude lower than in centrally located populations^37,38^. Surprisingly, the highest mitochondrial diversity was detected in the northernmost studied population, a situation also found in the White-tailed Eagle where the nordic Kola population was characterised by one of the highest diversity values^38^. The high diversity in White-tailed eagle was attributed to the high degree of admixture from various historical lineages^38^, and this may explain the unexpected pattern in the Greater Spotted Eagle. Also, the more recent population subdivision may have contributed to genetic diversity retention, as has been seen in the Mauritius kestrel^31^.

The two spotted eagle species hybridise extensively across Eastern Europe, and in some populations mixed pairs outnumber pure-species pairs^26^. Undoubtedly, introgression significantly affects the contemporary nuclear genetic architecture of the European Greater Spotted Eagle population, but in the current study all known hybrids were excluded from microsatellite and intron analyses. Although our methods reduced the effect of recent hybridisation on genetic diversity, we cannot exclude completely the influence of interbreeding on genetic diversity in the more distant past. However, relatively high diversity was detected also in mtDNA, which, due to its maternal heritability, is not affected by hybridisation. This confirms that high genetic diversity has been retained in the fragmented Greater Spotted Eagle populations, regardless of hybridisation, and, as of yet, there is no evidence of inbreeding depression.

Population structuring has been found in most raptor species that have been studied. Often this is attributable to the effect of isolation by distance—populations that are geographically more distant from one another are also genetically more different^18,37^. However, this does not seem to be the case for the Greater Spotted Eagle, where admixture of various genetic lineages may have undermined this tendency. High mobility amongst birds of prey facilitates gene flow across populations. As a result, genetic differentiation between populations is typically shallow, although genetic differences between populations are often significant^19,33–35,37^. Moreover, and in concurrence with our findings, admixing of two groups across an east-west gradient has been found in several Eurasian raptors^19,34,37–40^. We studied only marginal populations of a widespread species, which may be more differentiated from one another than from central ones^41^. More extensive sampling across Russia is needed to reveal population structure across the range. Moreover, in the (currently defined) monotypic Greater Spotted Eagle several morphologically distinguishable groups have been separated^42^, which suggest that some genetic clusters might exist. The inclusion of more samples from eastern parts of the range is necessary before final conclusions can be reached about the significance of population structuring.

In migratory species, regular long-distance movements play an additional role in boosting gene flow, and subsequently mitigating population structuring. Philopatry forces most individuals to return to their breeding grounds, but some birds, especially in saturated populations, may settle and breed in suitable habitats along the migration route^23^. In spotted eagles the possibility of this is strikingly illustrated by pairs breeding far from their main distribution range. For instance, a Greater Spotted Eagle has bred in Germany, far from their closest extant breeding population in eastern Poland^26,43,44^. Similarly, single pairs of Lesser Spotted Eagles have bred in France and Spain, and probably in Algeria^45–47^, when their closest populations are located in north-east Germany and Slovakia. In our study we identified no recent migrants from other populations. This might have been due to the rarity of such events, as philopatry has relatively strong effect in the declining remnant, and thus unsaturated, populations. Indeed, an Estonian Greater Spotted Eagle, having spent at least five years out of the current breeding range of the species in Scandinavia, returned to breed in Estonia (Ü. Väli, unpublished results from telemetry). However, a single immigration event could change significantly the genetic architecture of a population^22^.

Although seasonal migration may facilitate gene flow between populations, as described above, migration is probably even more deeply linked with population differentiation because different migration routes probably reflect historical postglacial colonisation routes^48^. Individual Greater Spotted Eagles breeding in one area can spend their non-breeding time in places that are distant from one another. For instance, birds from the Estonian and Polish populations we studied, winter across a huge longitudinal range from south-western Europe to the Middle East and north-east Africa^49–51^. The two groups detected by microsatellites in the current study may also be linked to different migration routes and destinations, and this may have also increased the differentiation found in the east-west gradient. On the other hand, settlement of individuals along the migration route before reaching to their natal areas may lower differentiation along the north-south gradient. However, the patterns detected in this study need further exploration, as nuclear splits have been suspected to result from recent demographic dynamics, whereas mitochondrial differentiation, which was not detected here, has been assumed to be a consequence of disjunct Pleistocene refugia^37^.

A star-like network and significantly negative Taijma’s *D* and Fu’s *Fs* values indicate that the European Greater Spotted Eagle population has undergone demographic expansion in the past^52^. Although the time estimations were rather variable, depending upon method and mutation rate, the expansion of European Greater Spotted Eagle population dates back to the late Pleistocene. Several European species have expanded since the Last Glacial Maximum, some 10000 years ago^53^. However, the expansion of the Greater Spotted Eagle might have an earlier genesis if it initiated in Asia where glaciers were more northern in their distribution and species’ ranges were less constrained. Interestingly, diversity levels among Greater Spotted Eagle populations were similar for microsatellites, but somewhat more variable for introns. It may be due to faster mutations for microsatellites, and thus a more rapid recovery after historical bottlenecks than for the slowly mutating introns.

It is more difficult to detect recent bottlenecks, especially in long-lived species, if population levels have been low during a relatively short time period^21^. Despite recently described population declines across most of Europe, the Garza-Williamson *M* index did not reveal any bottlenecks in the European Greater Spotted Eagles, and an excess of heterozygotes suggested only one local population decline in recent times. The bottleneck that was detected in Poland was suggested also by a subtle mode shift in allele frequency proportions. In fact, field observations corroborate the genetic indication of this population bottleneck, and suggest that the small Biebrza population is not just a remnant of a large population that has recently declined, but is the re-establishement of a population in the 1980s from few remnant pairs^54^.

The current study focused on the European part of the global population, which stretches across Eurasia. Further studies that are spatially more extensive (i.e. across the global distribution) would shed more light on genetic diversity and structuring among Greater Spotted Eagle populations. Moreover, although estimations of current global population size have been published, further validation of these estimates is needed^24^. As field studies of the wetland-dwelling Greater Spotted Eagle are labour intensive, non-invasive genetic methods are potentially a more cost-effective approach in estimating actual and effective population sizes^55,56^.

Declines and extinctions of raptor populations have given rise to several reintroduction and augmentation programmes^57^. For example, the declining Lesser Spotted Eagle population in Germany has been supplemented by translocating individuals from Latvia, where they are abundant^58^. Our study suggests that there is currently no need for genetic augmentation of European Greater Spotted Eagle populations because genetic diversity is not low, when compared to other, similar species. Instead, we recommend focusing on other conservation efforts, such as protection of nest sites, conservation of habitats and reducing poaching, which seem to be the major threats faced by this species^54^. However, translocations may be needed to increase current population sizes or re-establish populations in regions where they have gone extinct. Our results indicate that within Europe there are no restrictions on translocations from the genetic perspective as the differentiation between populations is low and there is ongoing gene flow between them. However, before any such actions are taken, other ecological, demographical and behavioural variables should be considered.

On the other hand, our results supporting management of a European Greater Spotted Eagle population as a single conservation unit^59,60^ should not be considered as advocating concentration of conservation actions on a single population. As is the case in the other species (e.g. White-tailed Eagle^21,37^), much genetic diversity is retained in small Greater Spotted Eagle populations, and these should be conserved as well.

## Methods

### Study species and sampling

In Europe, the largest population of the Greater Spotted Eagle occurs in Russia (600–800 pairs^24^) where, unlike seen in most published maps, the distribution is probably rather patchy (Ü. Väli & V. Dombrovski, unpublished data). Belarus harbours 120–160 pairs, mostly in the south^61^. Approximately 40 pairs breed in Ukraine^24^. A small population (≤15 pairs) occur in Poland, but only in the north-eastern part of the country^54^. Up to 30 breeding territories were found in Estonia in the late 20^th^ century^62^, but only a maximum of 10 territories remain^63^. There exists extensive hybridisation in Estonia, Poland and Belarus; the extent of hybridisation in Russia is largely unknown, although hybrids were detected in the only population in which it was studied in the Upper Volga^26^. Pure and hybridising pairs have been recorded also in Finland, Germany and Lithuania, but their numbers are low^26,43,64,65^.

The Greater Spotted Eagle is a solitary breeder; and pairs will reuse their nests year after year. Samples were collected during 1997–2012 in nearly all European countries, where the species breeds (Fig. 1). We collected blood and feather samples from nestlings and moulted flight feathers of adults during breeding season at nest sites. Sampling at nests ensured that our analyses were of local breeding populations, not vagrants or migrating birds. The greatest numbers of samples were collected in southern Belarus (Polessye region), north-eastern Poland (Biebrza valley) and Estonia. The population occurring in the Upper Volga region of Russia was also sampled. For the analysis of maternally inherited mitochondrial DNA (mtDNA), samples of single individuals were included from north-eastern Belarus, Latvia, Lithuania, south-eastern Poland, Ukraine, and from Germany, where single pairs have been found breeding far from the main breeding area^43,44^. Sample collection was carried out in accordance with relevant guidelines and national regulations and approved by the Genetic Bank of Wild Fauna at the State Scientific and Practical Center for Bioresources of the National Academy of Sciences of Belarus, Estonian Environmental Board and the director of the Biebrza National Park.

We avoided knowingly analysing samples from closely related individuals. If several samples were collected from a nest, we analysed either that of the nestling (in spotted eagles usually only one nestling fledges) or those of one or both of the adults. Although nestlings may have lower genetic diversity compared to adults, differences are only subtle^66^ and should not interfere with general patterns, especially as we sampled both age classes in all populations. Although the majority of moulted feathers originated from females because they attend the nest more than males^67,68^, we determined the sex of feather samples by analysing the CHD gene^69^. We excluded multiple samples from the same adults by analysing multilocus matches using GENALEX software, version 6.5^70^.

The Greater Spotted Eagle hybridises with the more common Lesser Spotted Eagle across the broad contact zone in Eastern Europe; the hybrids are viable and fertile^27^. Therefore we paid special attention to species determination prior to population-level genetic analyses. Species identity of the samples was initially confirmed by assignment analysis, where a large (*c*. 1500 inds.) Pan-Eurasian data set of Greater Spotted Eagles, Lesser Spotted Eagles and various types of hybrids between the two species was included^26,27^, Ü. Väli, unpublished data. The software NEWHYBRIDS version 1.1^71^ was used to calculate probability of Greater Spotted Eagle ancestry, *q*, for each individual using genotypes based on 24 microsatellite and 8 nuclear Single Nucleotide Polymorphism (SNP) markers (according to^26,27^). Only individuals with *q* values > 0.9 were included in further microsatellite and intron analyses. However, in the analysis of mtDNA we added also Greater Spotted Eagle-specific lineages from hybrid individuals. Comparatively, we conducted mtDNA analysis using only individuals identified as Greater Spotted Eagles, and obtained similar results.

### DNA extraction and selection of molecular markers

DNA was extracted using proteinase K treatment followed by phenol-chloroform purification or using an extraction kit (DNEasy Blood and Tissue Kit; Qiagen). When extracting DNA from the superior umbilicus of moulted flight feathers^72^, 100 mM dithiothreitol (DDT) was added to improve lysis.

Three types of genetic markers were analysed according to previously described protocols (Supplementary Table S1). Twenty-four microsatellites, developed from various eagle species^73–75^, were amplified and genotyped as described by^26,27^; 7734 nucleotides from ten introns of various nuclear genes^76,77^ were sequenced according to^27,77^; 470 nucleotides from the hypervariable pseudo-control region of the mitochondrial DNA (mtDNA) were sequenced following^29^. We also sequenced and conducted global analyses on longer fragments (764 nucleotides) from 40 individuals. The results of which were similar to those of our study samples.

No microsatellite genotyping errors, stutter peaks or allelic dropouts were detected by MICRO-CHECKER version 2.2.3 software^78^. In six microsatellite loci (Aa12, Aa27, Aa35, IEAAA05, IEAAA11, IEAAA05) evidence for null alleles was detected by MICRO-CHECKER. However, the results were consistent among populations for only IEAAAG11. Therefore, we removed this locus from further analysis, keeping the other five. We tested the effect of potential null alleles by estimating differentiation between populations using FreeNA software with and without ENA algorithm, which corrects for the bias induced by presence of null alleles^79^. No significant differences between the two sets of estimates were detected, which supports retaining loci for which the evidence of null alleles is inconsistent. Deviations from Hardy-Weinberg equilibrium were tested using ARLEQUIN version 3.5.2.2^80^, but no significant deviations were found after applying a Bonferroni correction for multiple comparisons. Significance of linkage disequilibrium between intron and microsatellite loci was tested using FSTAT version 2.9.3.2^81^ with 5000 permutations, but no significant linkage was found after applying a Bonferroni correction. Lack of linkage disequilibrium in the intron markers we used had been also verified by previous genomic studies^76,77^.

### Data analysis

Haplotypes of nuclear introns were reconstructed for each intron separately using the software PHASE version 2.1^82^. Number of haplotypes (Nh), haplotype diversity (H) and nucleotide diversity (π) for nuclear introns and mtDNA were calculated for all samples, and separately for each of the four populations from which we had more samples, using DNASP version 5.10^83^. In order to avoid loss of information (an Estonian mtDNA haplotype contained a 27-nucleotide deletion), gaps were excluded only in pairwise comparisons. Number of alleles, allelic richness (rarefactioned according to the minimum sample size, 5 individuals), observed and expected heterozygosities of microsatellites were obtained by ARLEQUIN and FSTAT. Between-population differences in allelic richness and heterozygosities were tested by analysis of variance.

Relationships between genotypes were initially explored by conducting principal coordinates analyses (using GENALEX) and factorial correspondence analyses (using GENETIX version 4.0.5.2)^84^. A median-joining network of mtDNA haplotypes was constructed with the software NETWORK version 5^85^. Differentiation between populations (pairwise *F*_*ST*_, distance calculated using the number of different alleles for microsatellites and pairwise differences for introns) was further evaluated using ARLEQUIN. Significance was determined by 1000 permutations of gene copies between individuals. ARLEQUIN was also used to study partition of genotypic diversity among and within populations (analysis of molecular variance based on computed distance matrix), and to conduct a Mantel test for the effect of isolation by distance. For the latter, geographical coordinates were determined as approximate middle points for each of the sampled populations.

At the individual level, population structure was further analysed by studying microsatellite data using the software STRUCTURE version 2.3.4^86^. The number of clusters (*K*) was detected with five independent simulations of potential *K* values up to four, using default parameters in the admixture model with correlated allele frequencies. Each run included 100,000 iterations for burn-in period, followed by 1,000,000 iterations for parameter estimation. Two approaches were used to choose the most likely number of clusters. First, number of clusters was estimated as a plateau-reaching values of log probability of data (*Ln P(D)* values^86,87^. Second, we calculated *ΔK* statistic, which is based on the rate of change in the log probability of data between successive *K* values; the correct number of clusters is the one with the highest *ΔK* value^88^. We assigned individuals to clusters using two approaches as per^37^ working on White-tailed Eagles. First, we used a strict 50% cut-off criterion, under which an individual is assigned to the cluster with higher membership; and second a more conservative 70% cut-off criteria, which retains individuals with intermediate values (for both clusters *q* = 31–69%) as intermediates between the two clusters. To identify recent migrants in the population, STRUCTURE was run employing a LOCPRIOR model, which uses information of the source population from which individual samples were taken.

To investigate the demographic history, we tested for signals of sudden population expansion in mtDNA using ARLEQUIN and DNASP. We calculated Tajima’s *D*^89^ and Fu’s *F*_*s*_^90^. Mismatch distribution was investigated and τ values for demographic and spatial expansions were calculated using ARLEQUIN. Time of the expansion was calculated as *t* = *τ*/2*ul*, where *u* is the haplotype mutation rate per site per million years and*l*is the number of studied nucleotides^91^. We used two mutation rates. First, 6%, given the 3-fold faster mutation rate in pseudo-control region in eagles, compared to the 2% of cytochrome b sequence^29^. Second, 14.8%, which is the mutation rate of the non-coding control region in avian mtDNA^92^, and which has been commonly used in earlier avian studies (e.g.^38^).

BOTTLENECK version 1.2.02^93,94^ was run to detect recent bottlenecks in the total sample and in each population separately. Statistical significance of the heterozygosity excess was performed by Wilcoxon sign rank test under a two phase mutation model (70% one-step and 30% multi-step changes; 1000 replications). The occurrence of a recent bottlenecks was evaluated also by checking mode-shift distortion in the distribution of allele frequencies^95^. Garza-Williamson indices, ratio of the allele number and the difference in allele lengths, were calculated using ARLEQUIN. Values above 0.82 have been found in populations that have not suffered a known reduction in size, and values below 0.70 have been detected in bottlenecked populations^96^.

## Supplementary information

Supplemetary Information

## Data Availability

The sequences analysed during the current study are available in the Genbank (accession numbers MK117281-MK117733). The dataset of microsatellite variation is available from the corresponding author on reasonable request.
