# Supplementary material for: High genetic diversity and low differentiation retained in the European fragmented and declining Greater Spotted Eagle (Clanga clanga) population
Source: Sci Rep. 2019 Feb 28;9:3064. doi: 10.1038/s41598-019-39187-1 (PMC6395815; doi:10.1038/s41598-019-39187-1)
Supplement: Supplementary file 1 — Supplemetary Information [file 41598_2019_39187_MOESM1_ESM.pdf]

Supplementary Information

High genetic diversity and low differentiation retained in the European fragmented and declining Greater Spotted Eagle (*Clanga clanga*) population

Ülo Väli, Valery Dombrovski, Marina Dzmitranok, Grzegorz Maciorowski, Bernd-Ulrich Meyburg

Table S1. List of studied nuclear markers

	Length	Na	Mean Ne	He	Ho	Reference
Introns						
ACP6	807	4	2.00	0.21	0.15	1
YME1L1	874	3	1.75	0.08	0.09	1
UBC6	669	3	1.75	0.07	0.07	1
ARHGEF9	713	5	2.75	0.28	0.30	1
PSMC1	951	9	4.25	0.62	0.74	1
PDCD11	706	9	4.00	0.50	0.41	1
BZW1	590	7	3.00	0.29	0.25	1
EIF3J	948	7	3.00	0.44	0.51	1
CSNK2A2	689	6	3.75	0.57	0.54	1
DDX42	787	2	1.25	0.08	0.40	1
Microsatellites						
Aa02	133_135	2	2.00	0.33	0.27	2
Aa12	123_141	10	4.75	0.44	0.36	2
Aa15	197_209	6	5.00	0.57	0.58	2
Aa26	146_170	9	5.50	0.58	0.61	2
Aa27	93_99	3	3.00	0.45	0.45	2
Aa35	249_267	9	7.25	0.75	0.67	2
Aa39	165_179	7	4.75	0.66	0.70	2
Aa43	112_132	10	6.75	0.76	0.74	2
Aa49	153–159	4	2.75	0.21	0.20	2
Aa53	130_144	7	5.75	0.72	0.73	2
Aa57	112_120	5	4.00	0.47	0.44	2
Hal1	116_120	2	2.00	0.41	0.41	3
Hal4	152_184	13	8.75	0.77	0.77	3
Hal7	143_155	5	3.75	0.56	0.57	3
Hal9	135_141	4	3.00	0.43	0.35	3
Hal13	150_154	3	2.25	0.31	0.33	3
Hal18	141_151	6	3.50	0.45	0.40	3
IEAAAG04	214_226	4	3.50	0.61	0.67	4
IEAAAG05	136_164	7	4.50	0.51	0.32	4
IEAAAG11	332–396	15	8.25	0.83	0.73	4
IEAAAG12	130_146	5	4.50	0.65	0.71	4
IEAAAG13	242_258	5	4.00	0.50	0.45	4
IEAAAG14	204_212	3	3.00	0.47	0.53	4
IEAAAG15	126_142	5	3.75	0.59	0.67	4

References. 1 - Backström, N., Fagerberg, S., Ellegren, H. 2008. Genomics of natural bird populations: a gene-based set of reference markers evenly spread across the avian genome. Mol. Ecol. 17, 964–980.; 2 Martínez-Cruz, B., David, V.A., Godoy, J.A., Negro, J.J., O'Brien, S.J., & Johnson, W.E. 2002. Eighteen polymorphic microsatellite markers for the highly endangered Spanish Imperial Eagle (Aquila adalberti) and related species. Mol. Ecol. Res. 2, 323–326.; 3 - Hailer, F., Gautschi, B., & Helander, B. 2005. Development and multiplex PCR amplification of novel microsatellite markers in the Whitetailed Sea Eagle, Haliaeetus albicilla (Aves: Falconiformes, Accipitridae). Mol. Ecol. Res. 5, 938–940.; 5 - Busch, J. D., Katzner, T. E., Bragin, E. & Keim, P. 2005. Tetranucleotide microsatellites for Aquila and Haliaeetus eagles. Mol. Ecol. Res. 5, 39–41.

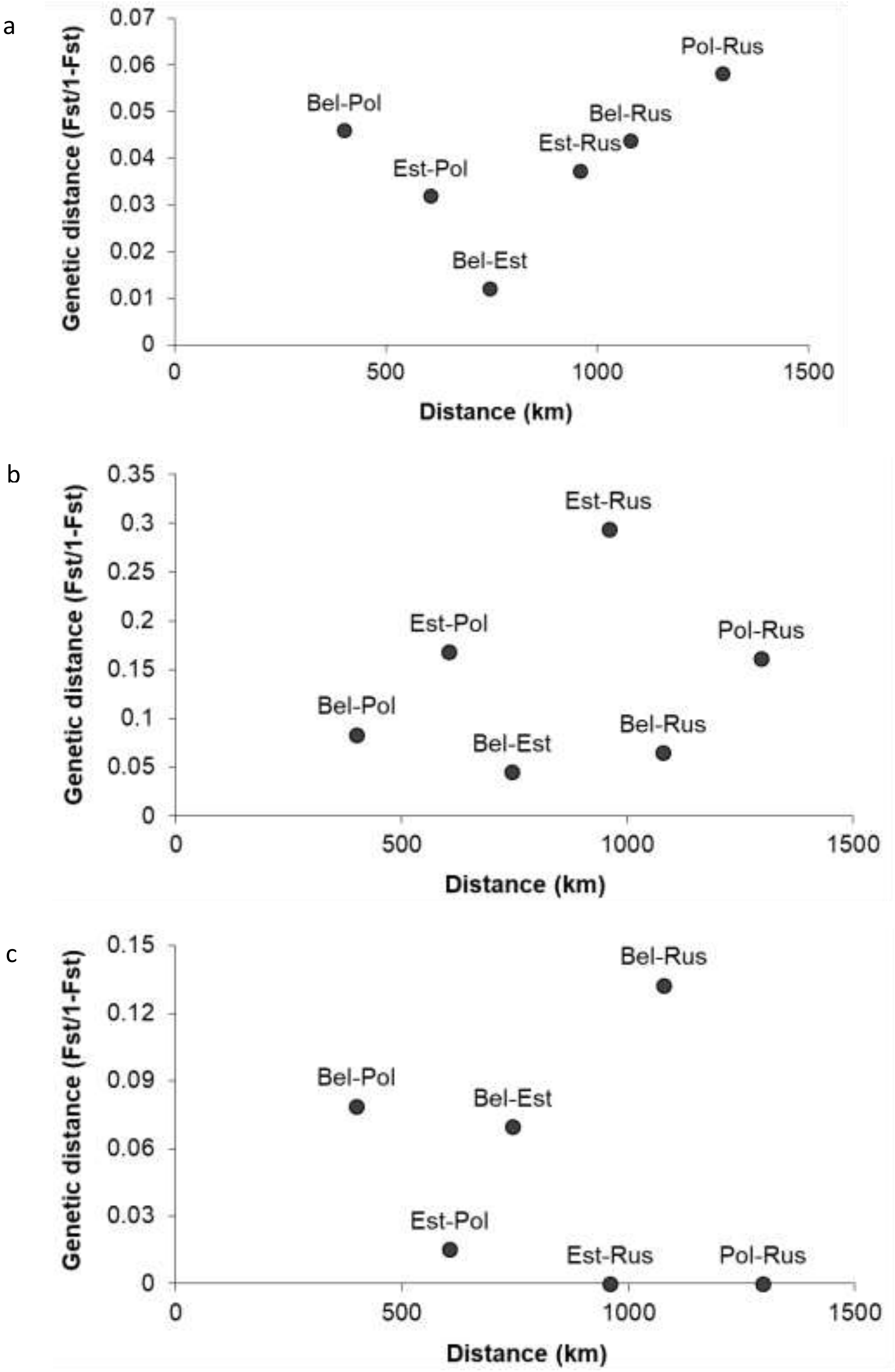

Figure S1. Relationships between genetic and geographic distances among four Greater Spotted Eagle populations using (a) microsatellites, (b) introns and (c) mtDNA

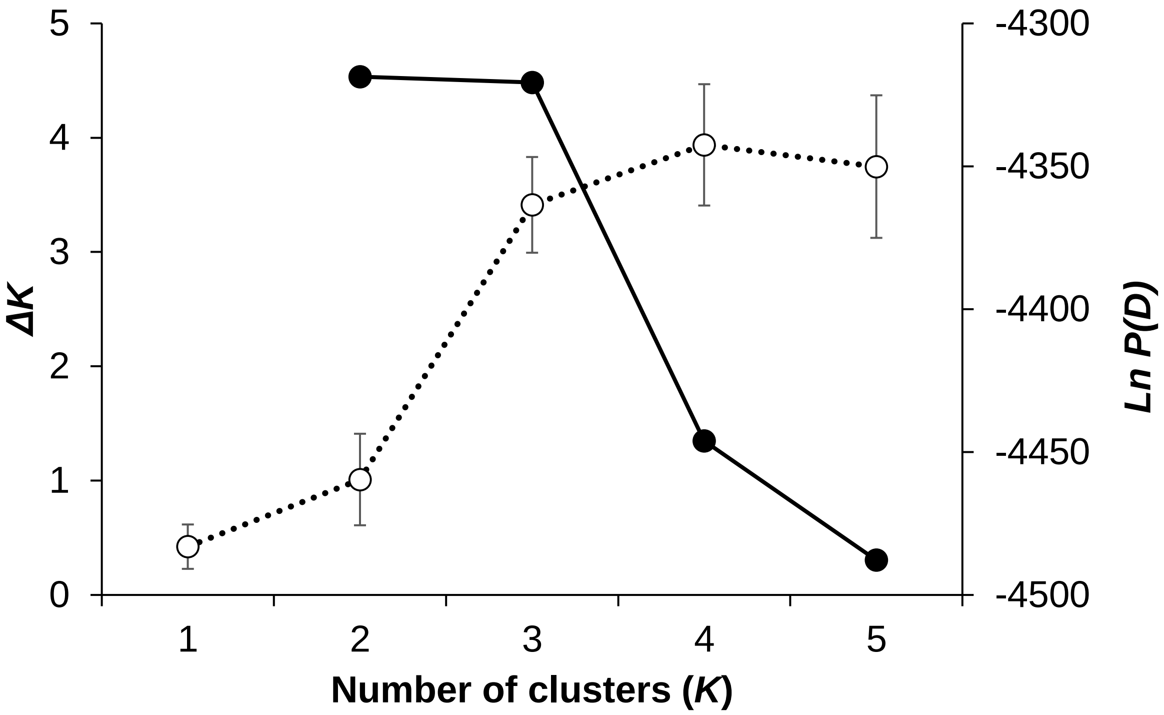

Figure S2. Suggested number of clusters according to *ΔK* (filled circles) and log posterior probability of K (*Ln (P(D)*; to ease comparison, absolute values are presented, empty circles) by the microsatellite analysis

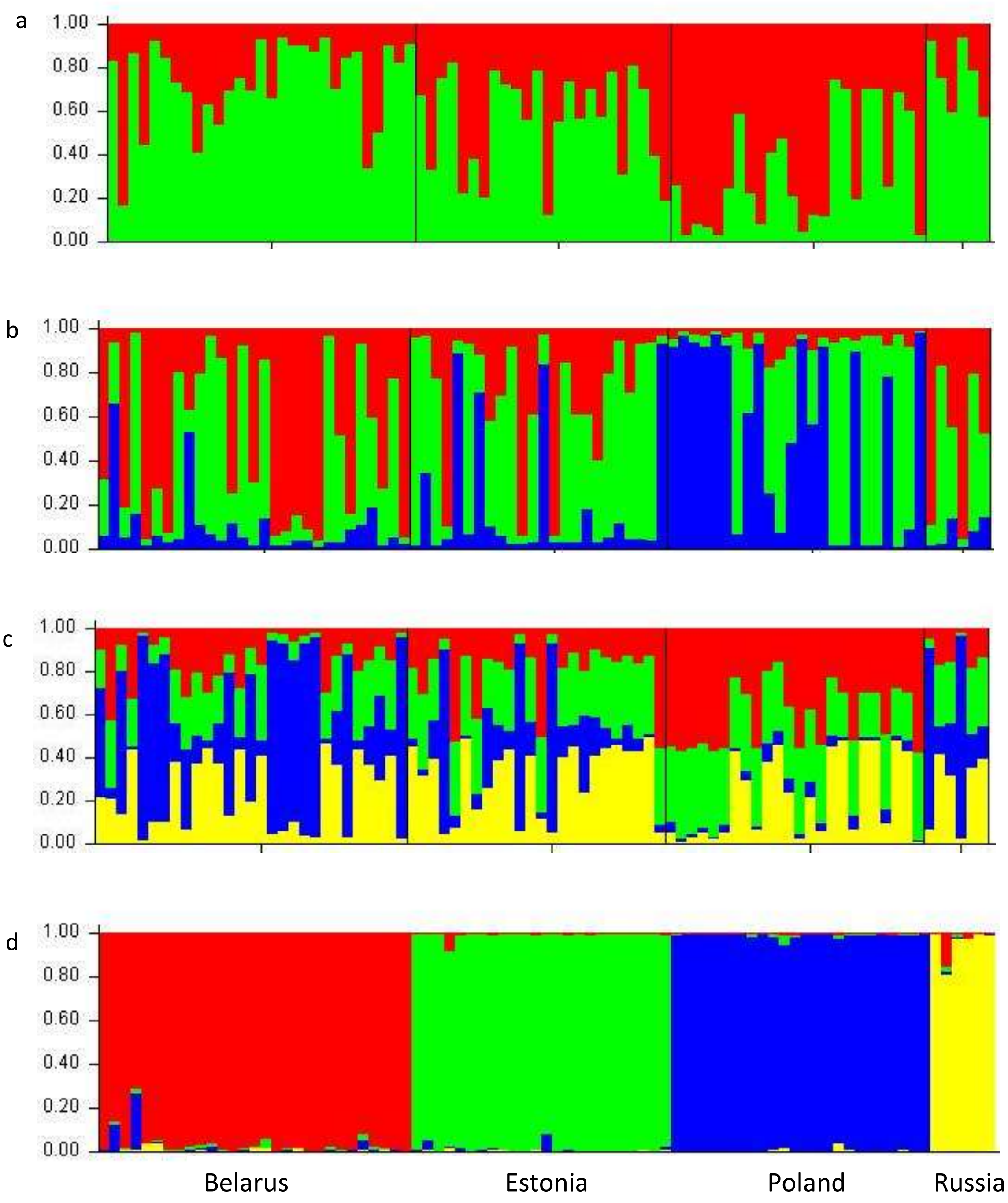

Figure S3. Assignment of individuals from four Greater Spotted Eagle populations into: (a) two, (b) three, or (c) four putative groups according to STRUCTURE analysis without prior information on actual population; and (d) detection of potential recent migrants by assignment of individuals into four putative populations with prior population information. The assignments are based on the of microsatellite genotypes.

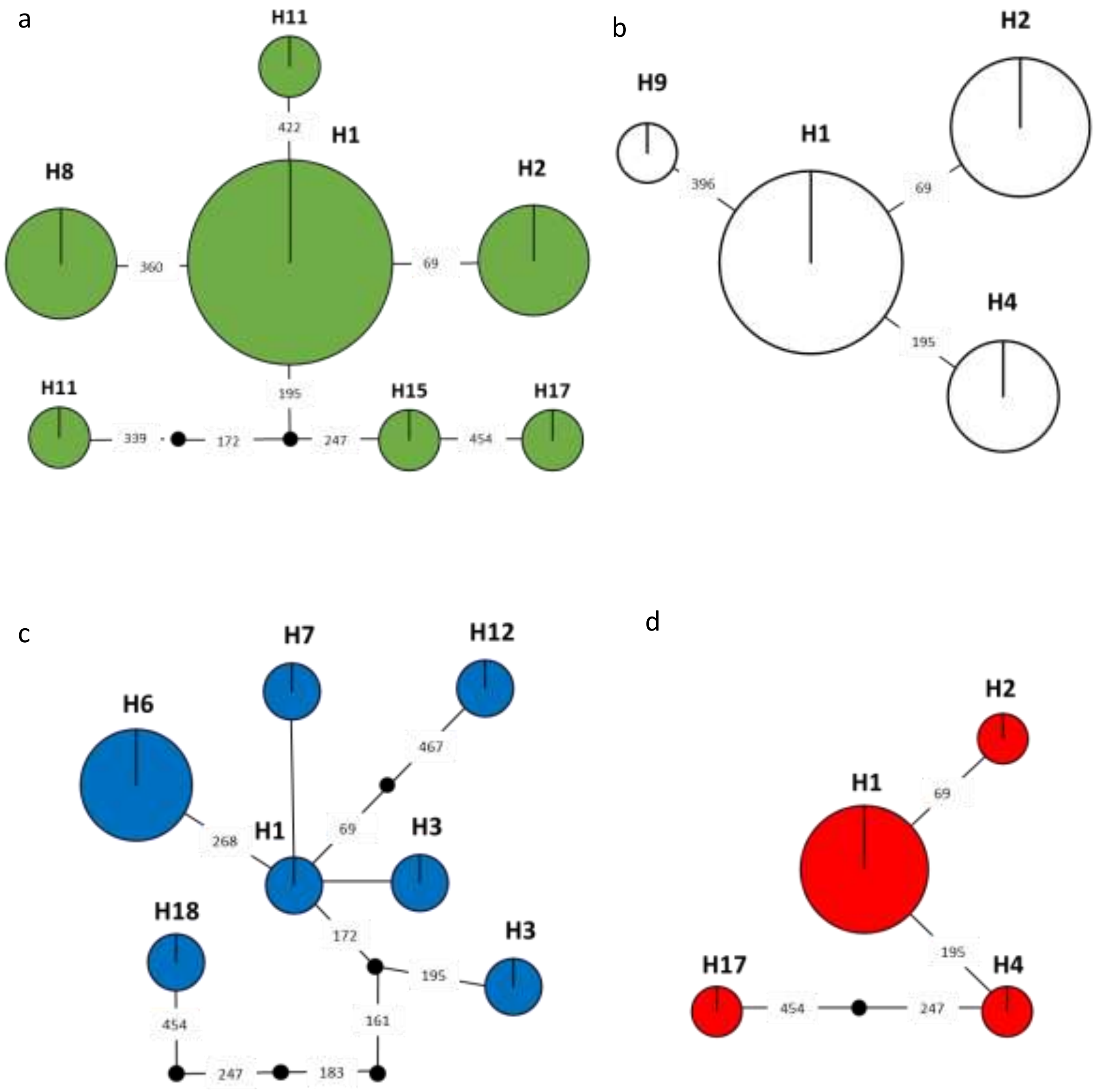

Figure S4. Median joining networks of mtDNA haplotypes from (a) Southern Belarus, (b) Poland, (c) Estonia and (d) Russia. Circle size is proportional to haplotype frequency. Haplotype labels are indicated as in Fig. 2, on connections defining mutations are presented. Indels were not included in the calculation of the network.

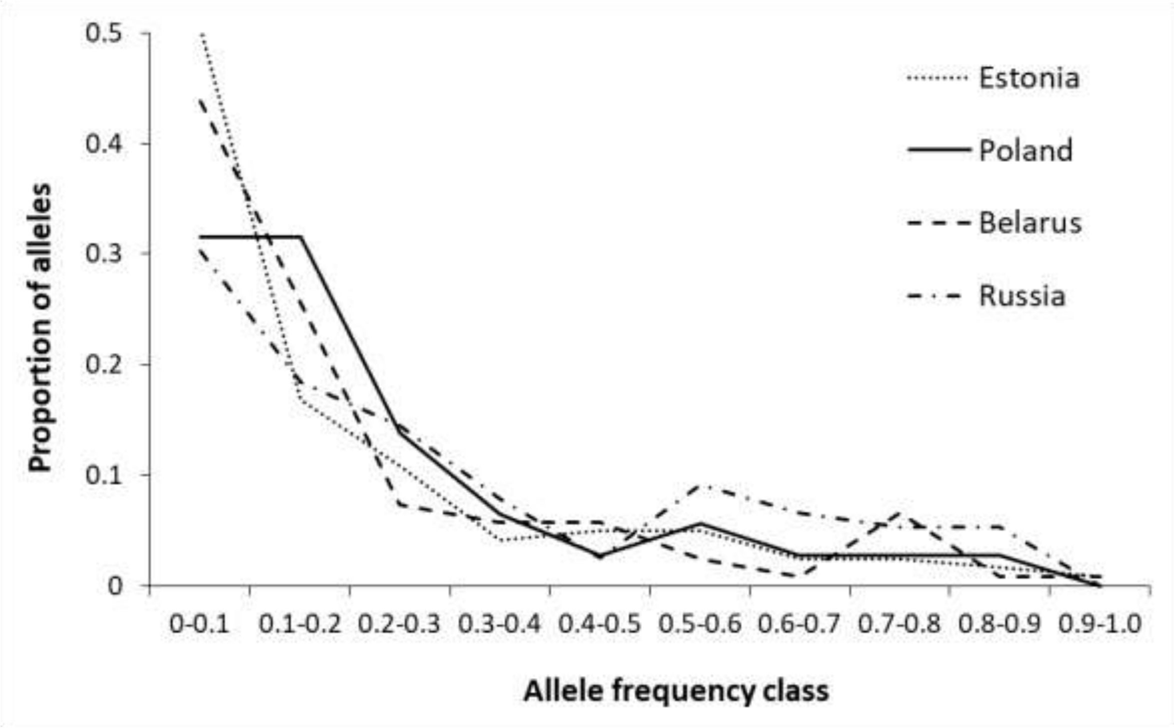

Figure S5. Microsatellite allele frequency distributions in four Greater Spotted Eagle populations.
